# Improvement in outpatient services using the WeChat calling system in the Shanghai Children’s Hospital

**DOI:** 10.12669/pjms.37.4.4301

**Published:** 2021

**Authors:** Dan Wu, Wenbin Cui, Xiulian Wang, Yanyan Huo, Guangjun Yu, Jinjin Chen

**Affiliations:** 1 Dan Wu, Shanghai Children’s Hospital, Affiliated to Shanghai Jiaotong University School of Medicine, Shanghai 200062, P.R. China; 2 Wenbin Cui, Shanghai Children’s Hospital, Affiliated to Shanghai Jiaotong University School of Medicine, Shanghai 200062, P.R. China; 3 Xiulian Wang, Shanghai Jiaotong University School of Medicine, Shanghai 200025, P.R. China. Shanghai Children’s Hospital, Affiliated to Shanghai Jiaotong University School of Medicine, Shanghai 200062, P.R. China; 4 Yanyan Huo, Shanghai Children’s Hospital, Affiliated to Shanghai Jiaotong University School of Medicine, Shanghai 200062, P.R. China; 5 Guangjun Yu, Shanghai Children’s Hospital, Affiliated to Shanghai Jiaotong University School of Medicine, Shanghai 200062, P.R. China; 6 Jinjin Chen Shanghai Children’s Hospital, Affiliated to Shanghai Jiaotong University School of Medicine, Shanghai 200062, P.R. China

**Keywords:** Incremental analysis, Time and Motion Studies, Technological Change, Innovation

## Abstract

**Objectives:**

We explored the utility of WeChat applet as part of the Outpatient Department (OPD) to provide patients with timely queuing information and compared it with the traditional calling system.

**Methods:**

Data for the WeChat calling system was extracted for the period of May 2018 to September 2018. Data for the traditional system was extracted for the same period from the year 2017. We compared the effective patient waiting time and nurse idle time i.e. nonproductive time spent on factors outside of employees’ control with the two systems. We also analyzed the relationship between the length of waiting time and conflicts between doctors and patients.

**Results:**

The mean wait time for the traditional calling system was 126 minutes, while the average idle time for nurses was 96 minutes/day. On the other hand, the mean wait time for the WeChat calling system was 33 minutes, and the average idle time for nurses was 72 minutes/day. The incremental profit (cost of traditional calling system – cost of WeChat calling system) achieved from switching systems was 13,879 yuan/month. Behavioral observations showed that wait time (OR=2.745, 95%CI 1.936~3.892 P<0.0001) was a risk factor for staff-patient conflict.

**Conclusion:**

The cost of the WeChat calling system was significantly lower than the traditional system. Also, the traditional calling system was time-consuming. Longer waiting time was the main factor affecting OPD quality and caused conflicts between doctors and patients.

## INTRODUCTION

Outpatient services are the primary medium through which a hospital provides services directly to the dependent population. The standard of the outpatient department (OPD) services offered by a hospital often determine the treatment quality, affordability and reputation of the healthcare center.^1^ In recent years, long waiting periods with the traditional OPD systems have had a great impact on the service quality and patient satisfaction.^2^,^3^ In order to maintain competitiveness, there is a need for hospitals to continually improve and review their service processes.

In the past decade, quality management systems (QMS), business process reengineering, and business process management (BPM) have emerged as important management tools which organizations can use to enhance business competitiveness.^4^ Similar models have also been applied in healthcare setups to reengineer the functionality of hospital OPD services.^5^ These changes have successfully reduced waiting times by at least 15%, while also increasing patient satisfaction, generating economic benefits, and enhancing organizational competitiveness.^6^,^7^ Indeed, reengineering can be considered as a radical revamp of system processes to achieve a comprehensive upgrade, including reducing costs and increasing service quality.^8^ Several studies in literature have undertaken a detailed cost-analysis of their hospital services and introduced measures to minimize costs while maintaining quality healthcare.^9^-^11^

WeChat software is a quick, convenient and easy to understand mobile software widely used in China.^12^ It not only has communication capabilities, but its multi-functional interface can also be used by third-party services to accept payment, provide queuing notifications, and several other functions.^12^ The integration of WeChat software in medical services can potentially provide several opportunities for innovation. WeChat can be used to schedule appointments, online consultations and also provide reminders for follow-up visits. Since long waiting time is more common in hospitals with large number of outpatient services, WeChat calling system can be suitably utilized in such setups for improving the OPD services, while it may not be suitable for smaller healthcare setups without high waiting times. If the number of outpatient service is small, In our institute, WeChat was integrated in the OPD service for better patient management in terms of waiting time. We hereby present a comparison of WeChat calling system and traditional calling system and assess the benefits of the software for reducing OPD waiting time and patient satisfaction.

## METHODS

**Fig.1:**
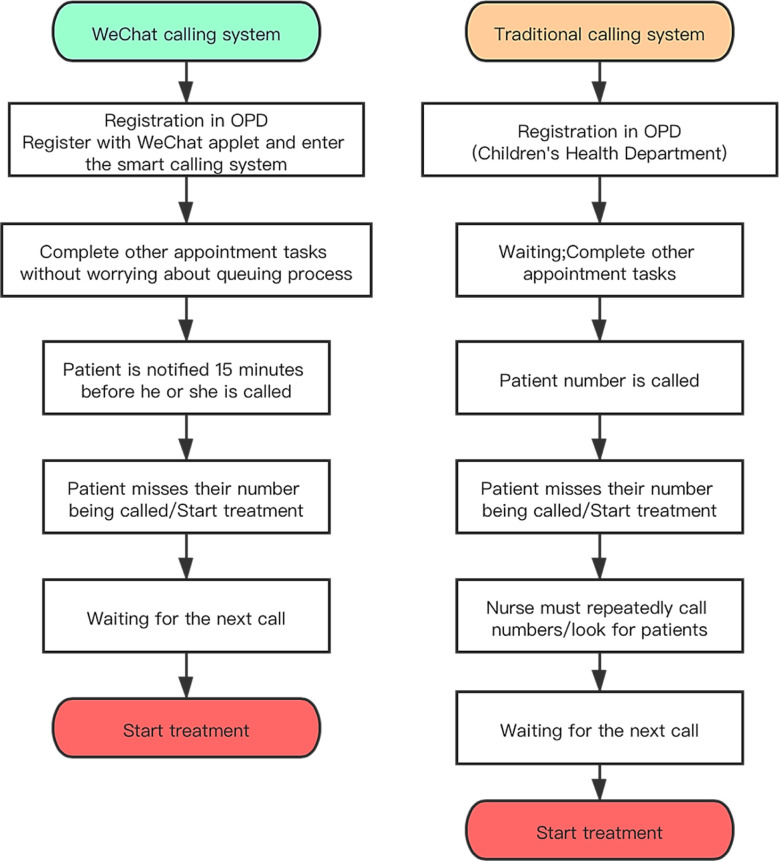
Process descriptions for the two systems. The left side of the figure shows the process of the WeChat calling system; the right is the traditional calling system. As illustrated, the overall procedure of the WeChat calling system is more concise.

The study was conducted in the Children’s Health Department of Shanghai Children’s Hospital, a teaching hospital attached to Shanghai Jiaotong University. The WeChat calling system was applied to the OPD in May 2018 and the research period was from May 2018 to September 2018. Data for the traditional system was extracted from the same period from the previous year (i.e. 2017) (Fig.1). Patients using the WeChat system directly register with the applet without queuing up for registration. Patients are then notified via the applet 15 minutes prior to their consultation. On the other hand, the traditional calling methods involved window registration with nurses calling out for patients prior to their turn for consultation.

### Wait time and idle time measurement

We compared the effective wait time for patients who used the WeChat calling system with the wait times of those who used the traditional calling system. We also compared nurse idle time for the two systems. Idle time was defined as nonproductive time spent on factors outside of employees’ control. The nonproductive time included time spent by workers in the work shift for work other than their own or invalid labor. For e.g. nonproductive time caused by the management included letting workers move materials, look for work stations, repair equipment and tools that are not related to their responsibilities, etc. The non-productive time caused by the individual included the time loss due to the worker’s own reasons, such as negligence of duty, unskilled activity, and temporary negligence, etc. By studying the exact amount of time spent in each activity for both systems, we estimate nurse idle time and patients’ effective wait times. “Wait time” usually began when the patient was first assigned a number and ended when the number was called. Nurse “idle time” is defined by the time spent “finding” patients who do not respond when their numbers were called. A time-motion study was used to determine patient wait times and nurse idle time in both systems.^13^,^14^

### Unit cost and incremental profit

In our study, the WeChat Calling System application did not generate additional service, material, or management costs. Therefore, the indirect cost comparison between the two systems is not reflected in the results. We calculated and compared the unit costs of each system by including labor costs (Cost × Number of personnel × Working time), costs due to idle time (Unit output × Number of personnel × Idle time), and other costs. Other costs included patent royalties (expenses for exploiting WeChat calling system) and taxes. Incremental profit was determined by the difference in cost between the traditional calling system and the WeChat calling system.

### Staff-patient conflict

Staff-patient conflict were recorded as harmful or even aggressive interactions between physician and patients. We observed whether any of the behaviors occurred during the waiting period to determine staff-patient conflict.

### Data analysis

We compared waiting time and idle time between the two groups using Students t-test. To estimate the unit cost, we designed a formula using Microsoft Office Excel software. We calculated the unit cost after inputting the cost data. Using SAS version 9.4 software to analyze data, a cost comparison was performed, comparing the total cost for each calling system. At the same time, we analyzed the relationship between the length of wait time and conflicts between doctors and patients.

## RESULTS

### Idle time and wait time

**Fig.2:**
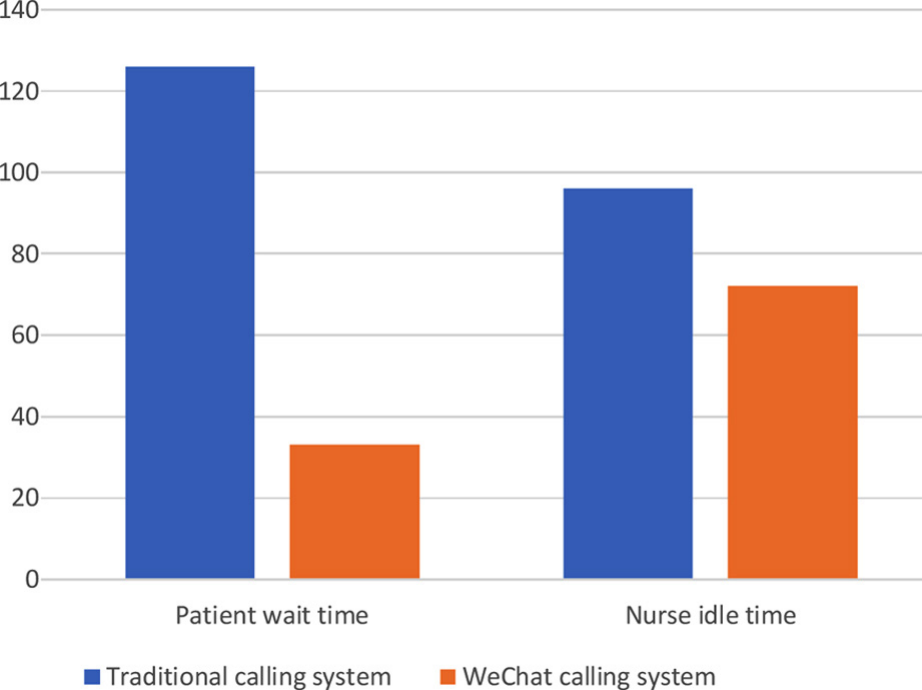
Idle time and wait time in the two systems The blue square represents the time taken by the traditional calling system, while the orange shows the WeChat calling system, can be seen from the figure, WeChat calling system was significantly reduced idle time and waiting time.

During the study, 45,740 patients visited the OPD service of the hospital and a total of 8,252 patients used the WeChat calling system. The mean wait time for the traditional calling system was 126 minutes, while the average idle time for nurses was 96 minutes/day. On the other hand, the mean wait time for the WeChat calling system was 33 minutes, and the average idle time for nurses was 72 minutes/day (Fig.2). Our analysis indicated statistical significant reduction in waiting time and idle time with the use of WeChat calling system.

### Unit cost and incremental profit

After the application of the WeChat calling system, the number of outpatient nurses was reduced from the original six posts to four posts, and the number of clerks increased by one. Tables 1 show unit costs and Table 2 the incremental profits. The labor cost in the traditional calling system was 48,000 yuan/month, while in the WeChat calling system was 35,500 yuan/month. The application of WeChat calling system reduced the unit cost of labor by 12,500 yuan/month. The costs due to idle time in the traditional calling system was 21,999 yuan/month, while in the WeChat calling system was 16,500 yuan/month. The WeChat calling system application reduced the unit cost of idle time by 5,499 yuan/month. Other costs associated with the traditional calling system were zero yuan/month, while in the WeChat calling system they were 4,120 yuan/month. The WeChat calling system application increased the unit cost by 4,120 yuan/month. The incremental profit (cost of traditional calling system – cost of WeChat calling system) achieved from switching systems was 13,879 yuan/month.

**Table-I:** Unit cost (per month) in the traditional and WeChat calling systems.

Labor cost		Rate(RMB)	Quantity	Working time(h)	Amount(RMB)
Traditional calling system	Nurses Clerk	45.455 /	6 /	176 /	48000 /
WeChat calling system	Nurses Clerk	45.455 19.86	4 1	176 176	32000 3500 35500
Idle time cost		Rate(RMB)	Quantity	Idle time(h)	Amount(RMB)
Traditional calling system	Nurses	104.16	6	35.2	21999
WeChat calling system	Nurses	156.25	4	26.4	16500
Other costs		Rate(RMB)	Quantity	Using time (day)	Amount(RMB)
Traditional calling system		-	-	-	-
WeChat calling system		187.27	1	22	4120

h, hours; RMB, yaun.

**Table-II:** Incremental profit (per month).

Cost items	Traditional calling system	WeChat calling system
Labor	48000	35500
Idle time-based cost	21999	16500
Other cost	/	4120
Total	69999	56120
Incremental profits	13879*	

(* Incremental profit 13879 ±703.68 t=44.10 P<0.01). All costs in RMB, yuan.

### Staff-patient conflict

**Fig.3:**
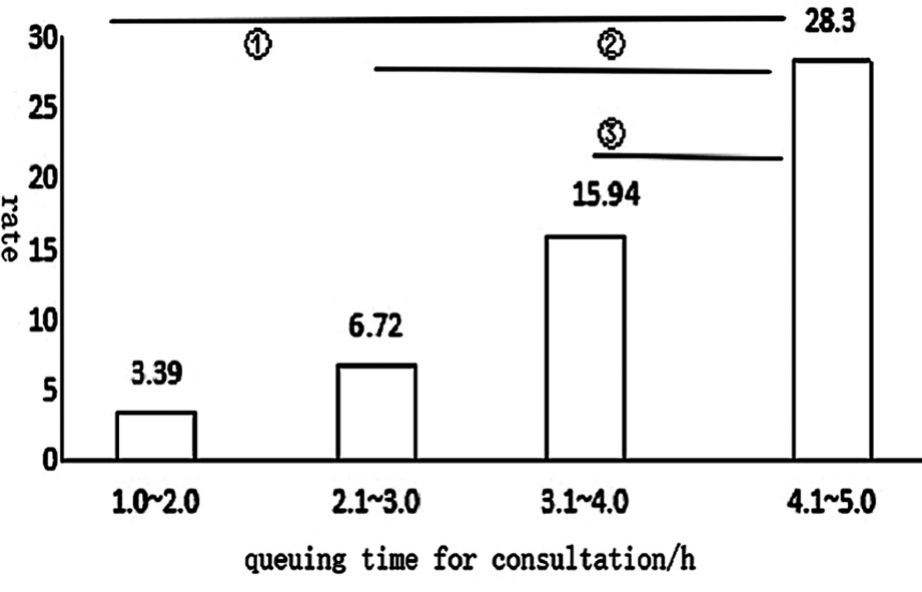
Comparison of waiting time and incidence of staff-patient conflict. Different wait times on x-axis and incidence of conflict on y-axis

After the quantitative process, we use a Logistic regression model (Tables III and IV; Fig.3) to analyze influencing factors, including gender, age, and wait time. The results showed that increased wait time was a risk factor for staff-patient conflict (OR=2.745, 95%CI 1.936~3.892 P<0.0001). It can be seen from Fig.2 that there was a variation in the incidence of conflict between doctors and patients with different wait times. There was a statistically significant difference in the incidence of conflict events between wait times of 4.1 to five hours and wait times of 2.1 to three hours and one to two hours (P<0.001). However, there was no significant difference in the incidence of events between wait times of 4.1 and five hours and those of 3.1 and four hours (P=0.053).

**Table-III:** Data of staff-patient conflict as per demographics and waiting time.

	Presence of conflict	Absence of conflict	z	P-value
Male/female	17/40	191/299	1.816	0.1778
Age (years)	33.54±4.687	32.52±4.746	1.537	0.125
Wait time (hours)	3.61±0.929	2.88±0.103	5.734	<0.0001

**Table-IV:** Logistic regression analysis model.

	β	OR	95%CI	P-value
Sex	-0.742	0.476	(0.242,0.936)	0.031
Age (years)	0.082	1.086	(1.016,1.161)	0.015
Wait time (hours)	1.010	2.745	(1.936,3.892)	<0.0001

OR, odds ratio; CI, confidence intervals.

## DISCUSSION

**Fig.4:**
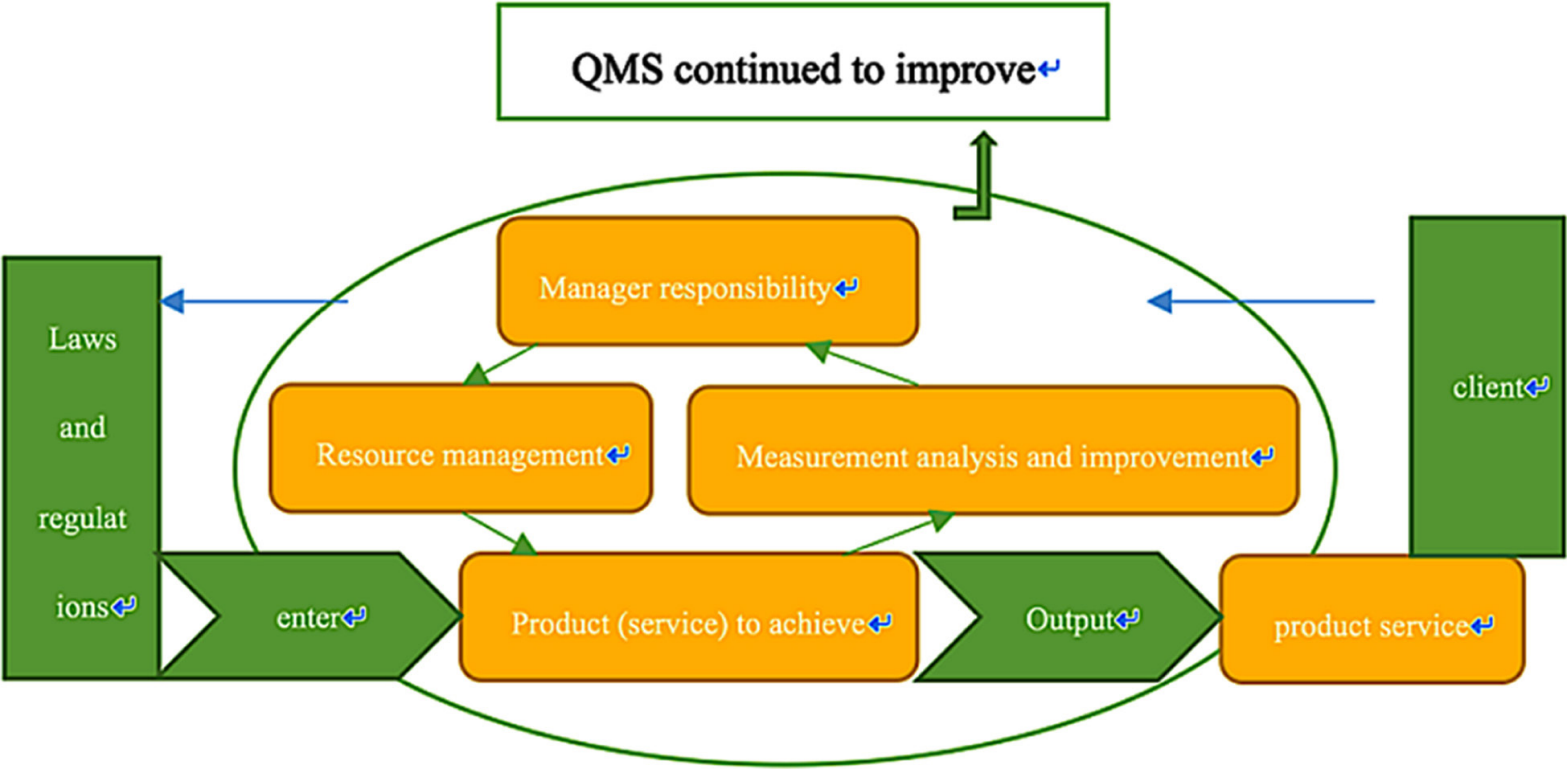
Quality management system (QMS). QMS is a management system used to direct and control an organization, and it provided the theoretical basis for our research.

In almost every industry, organizations have found that they can make significant improvements in their business performance and customer service by redesigning their business processes. These reforms require the freedom to change organizational structures and release core business processes from unnecessary additional activities. However, in the healthcare industry, medical personnel must comply with various legal regulations and treatment guidelines.^15^ In the face of these regulations, many institutes in the healthcare industry have focused on improving service and overall patient experience. With the deepening of China’s medical and health system reform, hospitals are facing increasingly fierce competition in the medical market. As the core of medical market competition, medical quality has directly affected the sustainable development ability of hospitals. With the rapid development of quality management in China’s public hospitals, modern hospital quality management theory has gradually formed a system. A Quality Management System (QMS) is a system that guides and controls the quality of an organization.^16^ There are several definitions of QMS, but, essentially, a QMS makes an organization perform better than before, as opposed to improving the profitability, efficiency, or customer orientation of an organization.^17^ That is to say, the QMS is a complex system that focuses on the quality of all processes and activities.^18^ Fig.4 explains this concept in detail.

Our research is based on the above mentioned theories wherein we studied the application of the WeChat calling system for outpatient services in the Children’s Health Department of Shanghai Children’s Hospital. We thought that by adopting the system and accepting patient feedback, we could further improve operations and create a virtuous cycle of system improvement.

**Fig.5:**
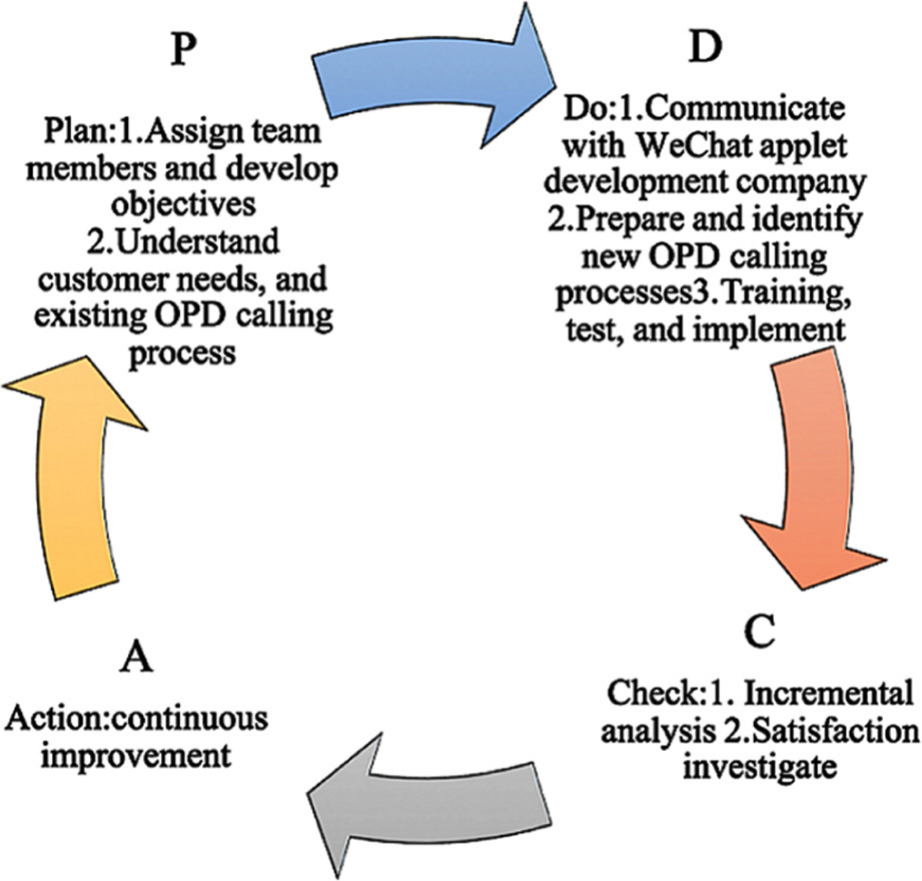
Deming Cycle (PDCA: plan–do–check–act). The four modules show the concrete steps of our research. The cyclical system shows the possibility of sustained improvement.

The two management tools, BPM and QMS, provide a general idea of how a management process can be transformed, and the specific implementation steps outlined in the Deming Cycle provide a framework for initiating a cycle of system improvement. The Deming Cycle is a management approach that controls and continually improves organizational processes; this includes the four part concept of ‘plan, do, check, act’ (PDCA).^19^ Fig.5 shows the Deming Cycle approach we used to implement the WeChat calling system in the hospital. First step is to ‘Plan’ where we assigned team members and developed objectives. Next, we attempted to understand client needs and existing calling processes. Second step is to ‘Do’ where we communicated with the WeChat applet development company to design a new OPD calling process. Then, we trained relevant staff and implemented the system. The third step is to ‘Check’. To do this, we implemented an incremental analysis and investigated satisfaction within the system. The last step is ‘Action’ through which we hope to achieve continuous improvement.

By analyzing outpatient check-in time and time of final consultation, we were able to determine average patient wait times. In addition, the average amount of time that nurses were idle was calculated by comparing the time nurses spent calling patients with the time they spent on patient care. We chose to examine nurse idle time, i.e., time not spent on patient care, because, studies have shown that increasing treatment time for nursing staff can increase economic benefits for a hospital.^20^ By reviewing financial statements, we were able to discern the cost of setting up the WeChat calling system and calculate the average total income of nurses, because the outpatient services run entirely by nurses in the department. Then, we appraised the unit costs associated with the hospital’s traditional calling system and the new WeChat system, and we performed an incremental analysis. Reducing idle time is an important factor in reducing overall costs, as increased idle time increases production costs.^20^

Through this study, it can be found that after the application of the WeChat calling system, nurse idle time is reduced by an average of 24 minutes per day. During the study period, the total cost savings were 89,995 yuan. The WeChat applet development costs and the related taxes and fees totaled 20,600 yuan. In the short term, the application of the WeChat calling system reduced unit costs, and the differences were significant. Assuming that the usage rate of the WeChat calling system is fixed, the cost increment will continue to decrease over time and the growth of economic benefits will become more obvious. In this process, the promotion of the WeChat calling system is especially important. Increasing the usage rate will further reduce direct costs.

The difference between the cost structure of the WeChat calling system and the traditional calling system was mainly reflected in the labor cost. The WeChat calling system reduced the labor costs in our institute. We believe both long-term and large-scale usage of the WeChat calling system can increase the time nurses spend treating patients and reduce nurse idle time. Studies have shown that increasing treatment time for nursing staff can increase hospital economic benefits and these studies provided support for our research.^20^,^21^ With regard to the problem of increased production costs due to idle time and the question of whether idle time increases as proficiency improves; addressing and solving these issues will be a subject of future research in our department.

The usage rate of the WeChat calling system was only 18% in our study, but the wait time for these patients was significantly shortened. Previous studies have shown that outpatient satisfaction depends on the quality of care and on wait time.^6^,^7^ Patient satisfaction is affected by many factors with varying complexity, but we believe, the WeChat applet can reduce patient dissatisfaction to some extent. While we did not analyze individual patient satisfaction due to the nature of the study, our study indicated that a short wait time could reduce the occurrence of conflict between doctors and patients. The application of WeChat applet provided a more reasonable and convenient visiting procedure for patients which reduced waiting time and the incidence of conflict between doctors and patients.

Our study has some limitations. Firstly, our research data is from a single hospital and currently only from a single health care department. Thus, our results cannot be generalized until further studies are carried out. Secondly, the outcomes of our study may depend on OPD load of a given department and we were unable to gauge how does the WeChat calling system function with excessive or comparatively lower OPD volumes. Thirdly, the applicability of WeChat calling system could be limited in individuals who are not well-versed with technology and this was not investigated in the current study.

In summary, our study shows that the application of WeChat calling system can reduce patient waiting time in the OPDs. It is reasonable to assume that the reduction in wait time and increased convenience of the system can improve patient satisfaction. The system also reduces labor costs and nurse idle time which can provide economic benefits to the health-care institute. Moreover, users are likely to enjoy the convenience and time-saving benefits, with the greatest possibility of reducing the possibility of staff-patient conflicts. We suggest that the WeChat calling system can be used in OPD and is worth promoting.

### Authors’ contributions:

**DW & WC:** Designed the project.

**XW& YH:** Did data collection and statistical analysis.

**DW & WC:** Did manuscript writing.

**JC & GY:** Edited the manuscript.

**JC & GY:** Take the responsibility and are accountable for all aspects of the work in ensuring that questions related to the accuracy or integrity of any part of the work are appropriately investigated and resolved.
